# Nanophase-Separated Copper–Zirconia Composites for Bifunctional Electrochemical CO_2_ Conversion to Formic Acid

**DOI:** 10.1021/acsami.3c02874

**Published:** 2023-05-04

**Authors:** Anna Strijevskaya, Akira Yamaguchi, Shusaku Shoji, Shigenori Ueda, Ayako Hashimoto, Yu Wen, Aufandra Cakra Wardhana, Ji-Eun Lee, Min Liu, Hideki Abe, Masahiro Miyauchi

**Affiliations:** † Department of Materials Science and Engineering, School of Materials and Chemical Technology, Tokyo Institute of Technology, Meguro, Tokyo, 152-8552, Japan; ‡ Uzbek-Japan Innovation Center of Youth, Tashkent 100095, Uzbekistan; § Department of Materials Science & Engineering, Cornell University, Ithaca, New York, 14853-1501, United States; ∥ National Institute for Materials Science, Tsukuba, Ibaraki, 305-0044, Japan; ⊥ Graduate School of Pure and Applied Sciences, University of Tsukuba, Tsukuba, Ibaraki, 305-8571, Japan; # Biofunctional Catalyst Research Team, RIKEN Center for Sustainable Resource Science, Wako, Saitama, 351-0198, Japan; ¶ Hunan Joint International Research Center for Carbon Dioxide Resource Utilization, School of Physical and Electronics, Central South University, Changsha 410083, Public Republic of China; ∇ Graduate School of Science and Technology, Saitama University, Saitama 338-8570, Japan

**Keywords:** nanophase separation, Cu_51_Zr_14_, Cu#ZrO_2_, electrochemical CO_2_ reduction, bifunctional catalysis, in situ Raman, formic acid

## Abstract

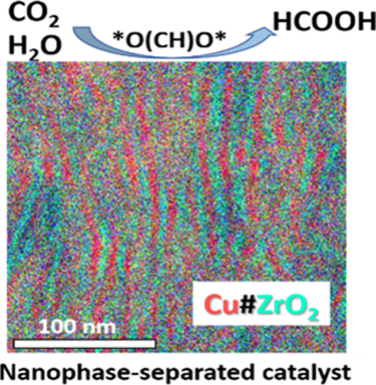

A copper–zirconia composite having an evenly distributed lamellar texture, Cu#ZrO_2_, was synthesized by promoting nanophase separation of the Cu_51_Zr_14_ alloy precursor in a mixture of carbon monoxide (CO) and oxygen (O_2_). High-resolution electron microscopy revealed that the material consists of interchangeable Cu and t-ZrO_2_ phases with an average thickness of 5 nm. Cu#ZrO_2_ exhibited enhanced selectivity toward the generation of formic acid (HCOOH) by electrochemical reduction of carbon dioxide (CO_2_) in aqueous media at a Faradaic efficiency of 83.5% at −0.9 V versus the reversible hydrogen electrode. In situ Raman spectroscopy has revealed that a bifunctional interplay between the Zr^4+^ sites and the Cu boundary leads to amended reaction selectivity along with a large number of catalytic sites.

## 1 Introduction

Dependence of humanity on fossil fuels and the resulted emissions of carbon dioxide (CO_2_) into the atmosphere created a loop, worsening the world environment. Much effort has been made to reduce the carbon footprint and mitigate the CO_2_ emissions as well as to diminish the role of unrenewable fossil fuels in industry. One of the most intensively explored routes is the direct conversion of CO_2_ into valuable chemical products including formic acid (FA). The outbreak of COVID-19 results in an increased commercial value of FA due to the high demands in poultry, textile, and pharmaceutical industries.^1^ Conventional FA production technologies are based on the hydrolysis of different formates, such as methyl formate, which suffers from poor waste management of toxic byproducts and huge necessity in water supplies.^2^ It is desirable to develop highly efficient and selective reaction catalysts that enable the direct conversion of CO_2_ to FA without passing through the hydrolysis processes.

Copper (Cu) is an active catalyst for direct CO_2_ conversion in terms of its moderate binding ability toward the *CO intermediate and its positive energy for *H adsorption.^3,4^ Cu-based catalysts showed a Faradaic efficiency (FE) of 77.1% for direct CO_2_ conversion to FA in aqueous media for hollow fibers and 82.4% for carbon-anchored Cu nanoparticles.^5,6^ However, such pristine Cu catalysts are poorly selective for FA production, leading to undesired generation of chemical species, including carbon monoxide (CO) and hydrogen (H_2_). One of the most promising routes to improve reaction selectivity is the use of metal–oxide interfaces that can promote only the target reaction. Indeed, zirconium oxide (ZrO_2_)-supported Cu nanoparticles (ZrO_2_/Cu) selectively catalyze the conversion of CO_2_ to ethylene (COE).^10^ ZrO_2_ possesses basic hydroxyl groups (OH) on the surface to efficiently adsorb CO_2_ and suppress hydrogen evolution.^7−9^ The COE reaction was promoted via a bifunctional mechanism comprising the molecular transfer of a *O(CH)O* intermediate, which was formed on the ZrO_2_ surface from adsorbed CO_2_, and spilt over to the Cu surface across the Cu–ZrO_2_ interface to generate C_2+_ products via an *OCCO* intermediate.^10^ The recent study by Li et al. showed that interfacial ZrO_2_ favored the stabilization and retention of Cu^+^ species in the CuO@ZrO_2_ catalyst, which resulted in increased *CO coverage and promoted coupling.^11^

Recently, nanophase separation of alloys has garnered considerable attention as an accessible and scalable path to discover a high density of catalytically active metal–oxide interfaces with a narrow size distribution.^12^ Nickel–yttrium oxide (Ni#Y_2_O_3_) and rhodium–cerium oxide (Rh#CeO_2_) were materialized from Ni_2_Y and Rh_2_Ce precursor alloys, respectively, to demonstrate the enhanced catalytic performances for the dry reforming of methane.^13,14^

Here, we report the use of nanophase separation of a copper–zirconium (Cu_51_Zr_14_) precursor alloy to obtain a nanocomposite catalyst comprising a metal–metal oxide interface of Cu and ZrO_2_ (Cu#ZrO_2_). The Cu#ZrO_2_ catalyst exhibited enhanced selectivity for the electrochemical CO_2_ reduction reaction toward FA production in aqueous media through the bifunctional interplay of Cu and ZrO_2_ across their interface. Spectroscopic analyses have shown that the CO_2_ admolecules on the ZrO_2_ surface spill over to the Cu surface across the interface and react with the OH species to generate FA via sequential reaction steps involving *CO_2_^–^ and *OC(H)O*.

## 2 Materials and Methods

### 2.1 Synthesis of Cu_51_Zr_14_ Alloy Precursor

An ingot of the Cu_51_Zr_14_ precursor alloy was obtained by arc-melting of Cu and Zr metals in an argon (Ar) atmosphere. For this process, Cu foil (99.99%, Nilaco Corporation) and Zr chunks (99.99%, Nilaco Corporation) were weighted in a molar ratio of 51:14 placed on a water-cooled copper hearth and subjected to a plasma arc torch. The Cu_51_Zr_14_ precursor alloy ingot was powdered with an agate mortar in air and sieved to adjust the size of particles to between 40 and 50 μm. The Cu_51_Zr_14_ powder was placed on a ceramic boat and heated at 400 °C for 12 h in a stream of mixture gases of carbon monoxide (CO)- and oxygen (O_2_) at a molar ratio of 2:1 and a total flow rate of 100 mL min^–1^. The blackish-gray Cu_51_Zr_14_ powder was converted into a dark-purple nanocomposite of metal Cu and zirconium oxide (ZrO_2_), i.e., Cu#ZrO_2_.

### 2.2 Characterization

The synthesized Cu#ZrO_2_ material and the Cu_51_Zr_14_ precursor alloy were characterized by powder X-ray diffraction (pXRD) over a 2θ range of 20–80° with a Rigaku SmartLab diffractometer equipped with a D/teX Ultra detector. The elemental composition and crystallographic structure of the samples were identified with scanning transmission electron microscopy (STEM, JEM-ARM200F, JEOL) imaging equipped with an energy-dispersive X-ray spectrometer (JED-2300, JEOL) operating at an acceleration voltage of 200 kV. A field-emission scanning electron microscope [JSM-7500F (JEOL)] was used to observe the external morphology of the materials. X-ray photoelectron spectroscopy (XPS) was performed after 5 min and 5 h of reaction using an ULVAC-PHI 1600 instrument with a monochromatic Al Kα source. The binding energies were corrected using a carbon (C 1s) signal at 284.80 eV.

Hard X-ray photoemission spectroscopy (HAXPES) measurements were performed at BL15XU of SPring-8 (Super Photon Ring 8 GeV, Hyo̅go Prefecture, Japan). The excitation photon energy and total energy resolution were set to 5.95 keV and 240 meV, respectively. The measurements were done at room temperature, and the pressure of the analysis chamber of HAXPES was 1.1 × 10^–7^ Pa.

### 2.3 Catalyst Preparation

Carbon paper with an average geometrical area of 2 cm^2^ was sonicated for 20 min consequently in ethanol and ultrapure water (Millipore Q), after which it was dried in air at 80 °C for 1 h. The catalyst ink was prepared by sonication of 5 mg of Cu#ZrO_2_ in a mixture of ethanol (440 μL) and ultrapure water (1785 μL) with an addition of 5 μL of 5% Nafion (Sigma-Aldrich). The prepared ink was then drop-casted onto carbon paper and dried in air at 80 °C for 1 h to ensure evaporation of ethanol. For the purpose of comparison of catalytic activities, we performed the same evaluation protocol on commercial Cu powder (Nilaco Corporation, average particle size: 30 μm), Cu_2_O powder (Nilaco Corporation), and a mixture of Cu- and yttria-stabilized zirconia (YSZ, 5.2% Y-doped, Sigma-Aldrich) in a molar ratio 51 to 14. YSZ was chosen as the control due to similarity of phases between ZrO_2_ in Cu#ZrO_2_ and YSZ. The catalyst loading was always 5 mg for any of the materials.

### 2.4 Electrochemical CO_2_ Reduction

Catalytic activity and selectivity of Cu#ZrO_2_ were evaluated in a custom-made H-type cell that was filled with electrolyte solution of 0.1 M KHCO_3_ (CO_2_ saturated, pH 6.8). A silver–silver chloride (Ag/AgCl) electrode and a platinum wire were used as the reference electrode (RE) and counter electrode (CE), respectively. The reaction selectivity was evaluated based on the Faradaic efficiency (FE) that was calculated as follows
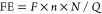
1where *F*, *n*, *N*, and *Q* are the Faradaic constant, number of electrons involved in the reaction, amount of the product in moles, and the total charge that flows between the working and counter electrodes, respectively. Gas product identification was performed using a gas chromatograph equipped with a dielectric-barrier discharge ionization detector (Shimadzu Tracera 2010). A proton nuclear magnetic resonance spectrometer (Bruker UltraShield Plus, 400 MHz) was used for the qualification of liquid products.

### 2.5 In Situ Spectroscopic Analyses by FT-IR and Raman Spectra

Two sets of measurements were conducted for obtaining diffuse reflectance infrared Fourier transform (DRIFT) spectra. For each of the experiments, a fresh catalyst sample was used, and either nitrogen (N_2_) or CO_2_ was bubbled through deuterium water (D_2_O) for 20–30 min prior to measurements.

In situ Raman spectroscopy was performed using a custom-made steady-flow setup (Figure S1). CO_2_-saturated 0.1 M KHCO_3_ solution is made to flow through a closed chamber with a transparent upper cover from the IN to OUT direction. A working electrode, a RE, and a CE were connected to a flow reactor, and the bias potential was applied from the open-circuit potential (OCP) to −1.0 V vs the reverse hydrogen electrode (RHE). CO_2_ gas was continuously bubbled through the electrolyte solution of 0.1 M KHCO_3_ from 20 min before the experiments onward to keep the setup always saturated with CO_2_. The gaseous products of reaction are removed from the chamber with flow of liquid.

## 3 Results and Discussion

**Figure 1:**
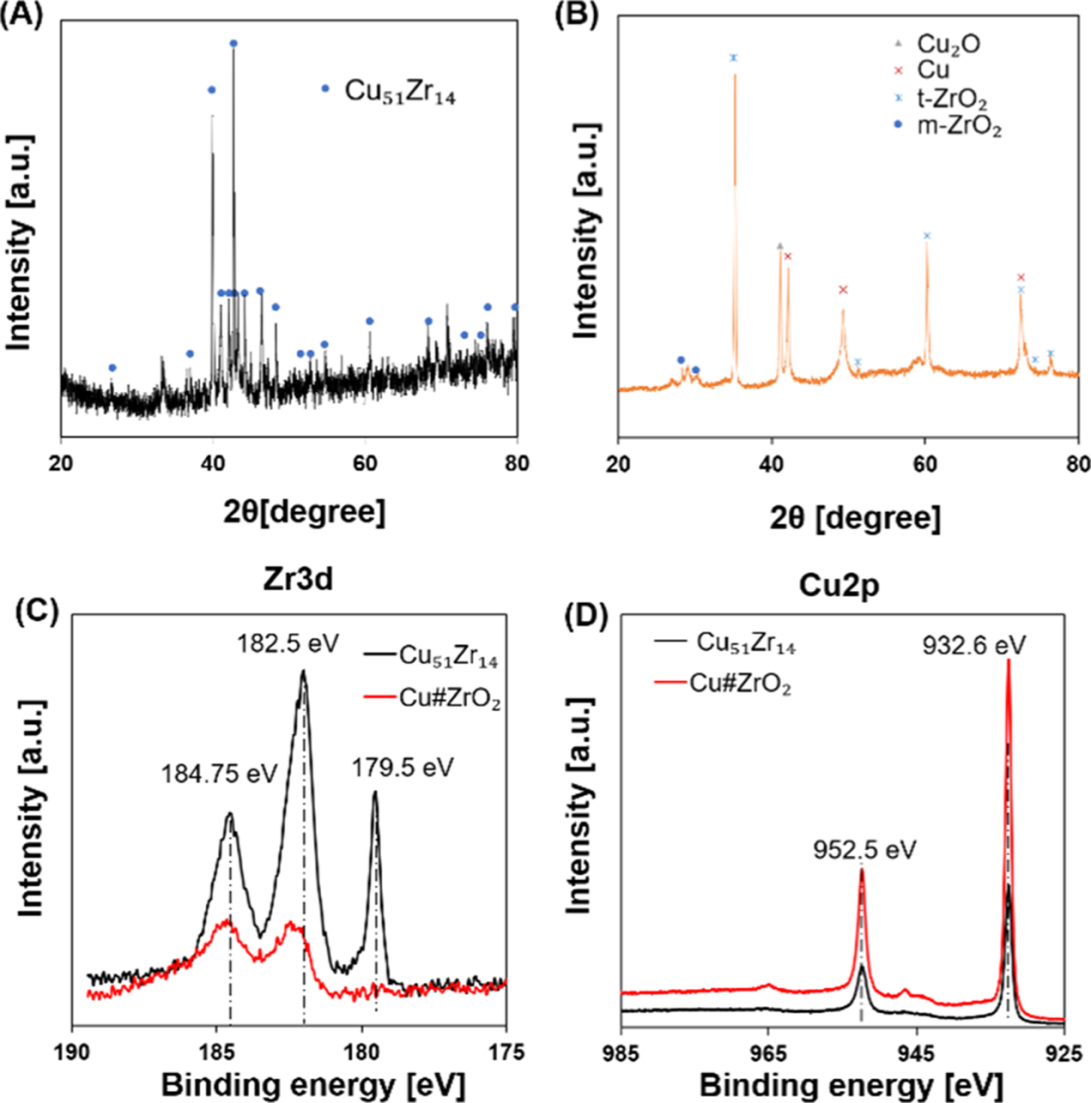
pXRD patterns of the Cu_51_Zr_14_ precursor alloy (A) and Cu#ZrO_2_ (B). HAXPES profiles of the Cu_51_Zr_14_ precursor alloy (black) and Cu#ZrO_2_ (red) in the Zr 3d region (C) and the Cu 2p region (D).

The pXRD pattern for the alloy precursor (Figure 1A) indicates that the main phase of the precursor was Cu_51_Zr_14_ (*P*6/*m*, *a* = 1.12444 nm, *c* = 0.82815 nm, α = β = 90°, γ = 120°)^15^ containing Cu_5_Zr (*F*4̅3*m*, *a* = 0.68700 nm, α = β = γ = 90).^16^ The pXRD pattern for Cu#ZrO_2_, shown in Figure 1B, indicates that the prepared Cu#ZrO_2_ mainly consisted of metallic Cu (face-centered cubic, *Fm*3̅*m*; JCPDS no. 85-1326) and tetragonal ZrO_2_ (t-ZrO_2_, *P*4_2_/*nmc*) with an inclusion of Cu_2_O (*Pn*3̅*m*)^17^ and monoclinic ZrO_2_ (m-ZrO_2_, *P*2_1_/*c*).^18^ Note that most of the ZrO_2_ phase in Cu#ZrO_2_ crystallized in a tetragonal form (t-ZrO_2_), which is thermodynamically less favorable than the monoclinic form (m-ZrO_2_). Figure 1C,D shows the HAXPES profiles of Cu_51_Zr_14_ and Cu#ZrO_2_. The HAXPES profile of Cu#ZrO_2_ in the Zr-3d core region is consistent with the reported data of ZrO_2_ (Figure 1C).^19^ The Zr^0^ 3d_5/2_ emission peak positioned at 179.5 eV on the HAXPES profile of Cu_51_Zr_14_ was not visible on that of Cu#ZrO_2_, indicating that the metallic Zr^0^ in Cu_51_Zr_14_ was fully oxidized to ZrO_2_ in Cu#ZrO_2_.^20,21^ Moreover, HAXPES has confirmed that the Cu phase in Cu#ZrO_2_ retained the metallic state of Cu^0^, the same as in the Cu_51_Zr_14_ precursor alloy (2p_3/2_: 932.6 eV; 2p_1/2_: 952.5 eV) (Figure 1D).

**Figure 2:**
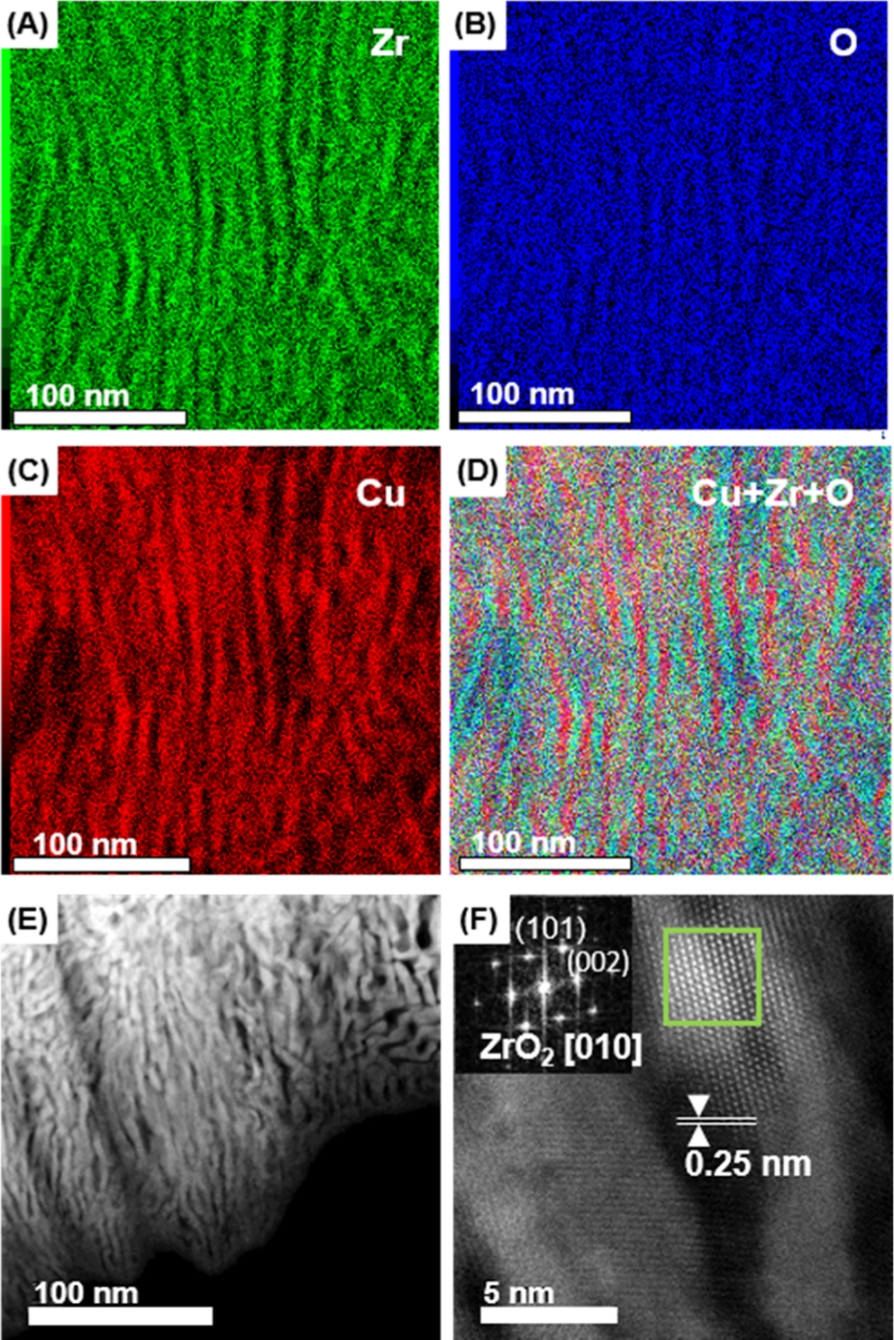
STEM–EDS images of the Cu#ZrO_2_ catalyst with a scale bar of 100 nm. Elemental mappings acquired at Zr L (A), O K (B), Cu K (C), and overlapped elemental mapping (D). ADF-STEM image showing a lamellar structure in Cu#ZrO_2_ (E). High-resolution ADF-STEM image at the Cu–ZrO_2_ boundary and an FFT pattern from the green square area (the inset) (F).

For the investigation of the nanostructure of Cu#ZrO_2_, we performed cross-sectional STEM (Figure 2). Elemental mapping images were first acquired with energy-dispersive spectrometry (EDS) for each of the constituent elements such as Cu, Zr, and O. All the STEM–EDS images show a lamellar texture with an average thickness of 5 nm. The Zr- and O species show the same special distributions as part of the ZrO_2_ phase in Cu#ZrO_2_, where the Cu species are exclusively distributed to the ZrO_2_ phase (Figure 2D).

An annular-dark field STEM (ADF-STEM) image of Cu#ZrO_2_ near the surface boundary shows that the lamellar structure consists of dark- and bright-contrasted phases (Figure 2E). High-resolution ADF-STEM on the bright-contrasted phase demonstrates a long-range atomic ordering (Figure 2F). A fast Fourier transform (FFT) pattern calculated for the area in the green square box in Figure 2F is assigned to the ⟨010⟩ zone axis of the tetragonal zirconia (t-ZrO_2_) (Figure 2F inset), which indicates that the bright-contrasted phase consists of t-ZrO_2_. The neighboring, dark-contrasted Cu phase shows an atomic fringe of the Cu(110) plane with a spacing of approximately 0.25 nm.

Figure S2 in the Supporting Information shows that Cu#ZrO_2_ exhibited larger current densities under CO_2_ bubbling than those under an Ar atmosphere (Figure S2A). The changes in current densities in a potential range more anodic than −0.3 V were attributed to a charging caused by the adsorption of CO_2_ molecules on the catalyst. Results of cyclic voltammetry shown in Figure S2B indicate the absence of self-oxidation of the catalyst during the electrolysis.

**Figure 3:**
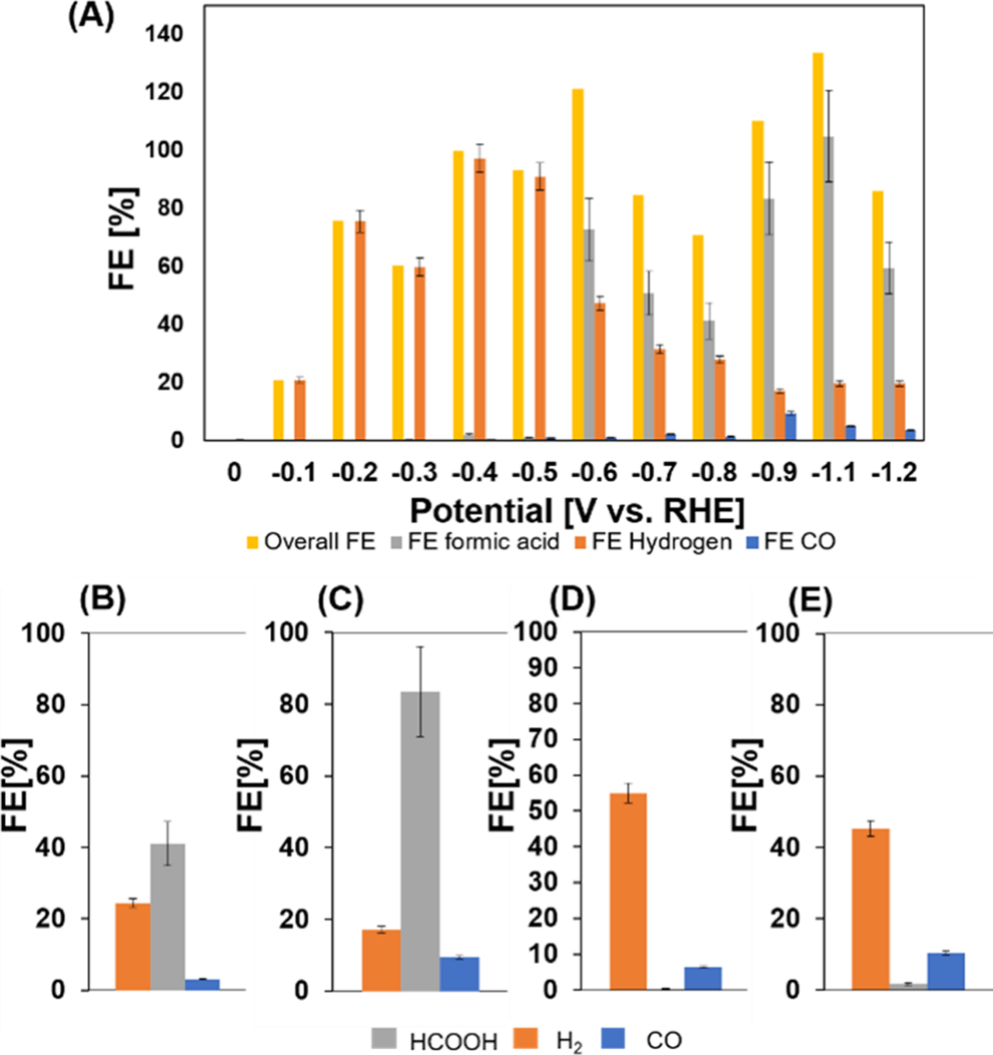
Product distribution of electrochemical CO_2_ reduction over Cu#ZrO_2_, quantified at the time after 3 h of electrolysis at −0.9 V vs RHE (A).Performance of electrochemical CO_2_ reduction over a mixture of Cu + YSZ (B), Cu#ZrO_2_ (C), commercial Cu (D), and Cu_2_O (E).

Figure 3A shows the FE of Cu#ZrO_2_ for CO_2_ reduction at different bias potentials. As indicated by the error bars, the FE values were scattered, but the total FEs were close to 100% under sufficiently negative potentials over −0.4 V vs the RHE. At potentials of more positive than −0.6 V, the major share of FE was occupied by hydrogen evolution. In contrast, at potentials more negative than −0.6 V, FA became one of the most predominant products: the corresponding FEs were 72% (partial current density: 2.4 mA/cm^2^) and 83.5% (partial current density: 16 mA/cm^2^) at −0.6 and −0.9 V, respectively. Hydrogen evolution was only 17% at −0.9 V, which is attributed to the lower hydrogen production selectivity of ZrO_2_ as reported by Soloveichik^9^ and Li.^11^ The other detected products were trace amounts of CH_4_ and C_2_H_6_, constituting less than 1% of FE each as shown in the Supporting Information (Table S1). Based on the result from DFT calculations by Xiao et al.,^22^ we can attribute these to enhanced *CO adsorption and dimerization on the Cu^+^/Cu^0^ pair. We also measured the partial current densities for the FA production (*j*_form_) over Cu#ZrO_2_ and control catalysts including Cu powder and a powder mixture of Cu- and yttria-stabilized ZrO_2_ (Cu + YSZ) (Supporting Information, Figure S3A) since the present ZrO_2_ structure in Cu#ZrO_2_ is similar to that of YSZ. Among those catalysts, Cu#ZrO_2_ exhibited the largest *j*_form_ at all given potentials. Moreover, Tafel analysis (Figure S3B) showed a slope of 155.9 mV/decade for synthesized Cu#ZrO_2_, which is smaller than those of Cu + ZrO_2_ and Cu, indicating faster reaction kinetics for CO_2_ reduction of our Cu#ZrO_2_ catalyst. We also confirmed the absence of reaction products over Cu#ZrO_2_ under the argon (Ar) atmosphere, indicating that the products in the CO_2_ atmosphere were purely from CO_2_ reduction.^23−25^ Overall, these results, while being comparable to recent Cu-and Bi-based catalysts, provide valuable insights at interface-related reactions, as shown in the Supporting Information (Table S2).^26,27^

Figure 3B–E shows FEs for FA, H_2_, and CO over the different catalysts at the time after 3 h of electrolysis at −0.9 V vs RHE. The FE for FA generation over the Cu#ZrO_2_ and Cu + YSZ catalysts reached 80 and 40%, respectively, whereas neither Cu nor Cu_2_O catalysts promoted FA generation. The results showed that the simple mixing of Cu and YSZ is not sufficient to achieve a high FE for FA generation. For the purpose of catalyst characterization after exposure to reaction conditions, FE-SEM and STEM–EDS images were taken after 3 h and 30 min of the electrocatalysis (Figures S4 and S5A–E). Figure S5 indicates the retention of the ZrO_2_ phase near the surface of the catalyst grain. Existence of ZrO_2_ even after cathodic bias application was further confirmed by XPS (Figure S6) and X-ray fluorescence analyses (Table S3). These results indicate that the catalyst surface is re-constructed at the initial bias application to a Cu-rich nanoporous structure that comprises a stable electrocatalysis center after several hours to stably produce FA. The results of 10 h of the stability test are shown in our Supporting Information (Figure S2C), and we could see the increase of current densities in the initial 4 h of electrocatalytic reaction, which is attributed to the ZrO_2_ reduction. After 4 h, the current densities became stable. FE-SEM images, taken before and after the CO_2_ reduction reaction, are shown in the Supporting Information (Figure S4).

**Figure 4:**
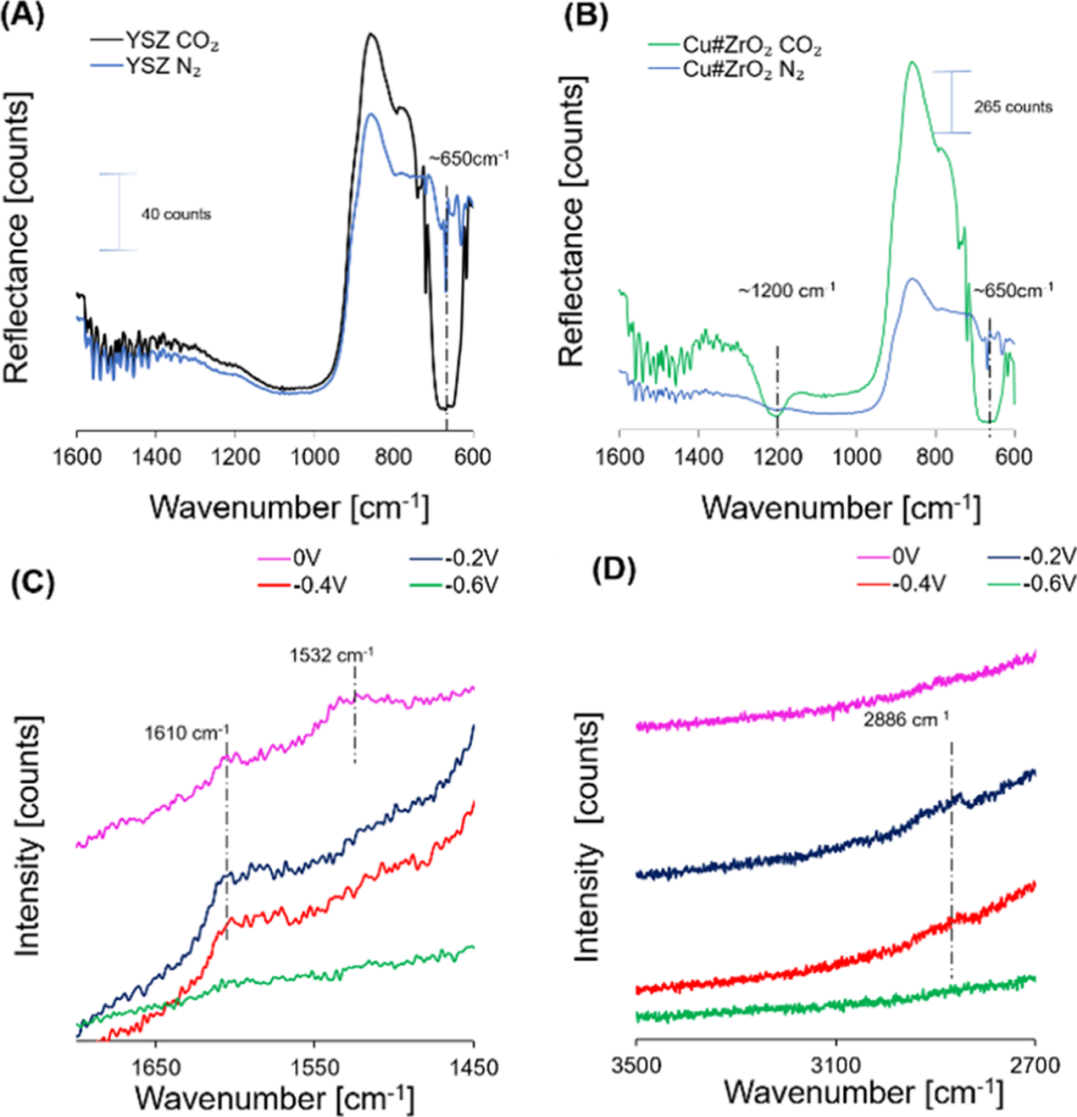
DRIFTS spectra recorded on YSZ (A) and Cu#ZrO_2_ (B) under D_2_O–CO_2_ adsorption. In situ Raman spectra recorded during electrochemical CO_2_ reduction at 0.0 V (purple), −0.2 V (blue), −0.4 V (red), and −0.6 V vs RHE (green) over Cu#ZrO_2_ in CO_2_-saturated 0.1 M KHCO_3_ (C,D).

In order to investigate the role of Cu- and ZrO_2_ sites in the molecular adsorption on initial stages of the FA generation reaction, we performed a set of DRIFTS over Cu#ZrO_2_ and YSZ in the atmosphere of deuterium oxide (D_2_O) vapor and CO_2_ (Figure 4A,B). The D_2_O vapor atmosphere is useful since it provides a high signal-to-noise ratio in the region of CO_2_-related species.^28,29^ The results indicate that the broad DRIFTS band around 650 cm^–1^ is attributed to the physisorbed linear CO_2_,^30^ which is recognized on both the control YSZ and Cu#ZrO_2_ under a CO_2_ atmosphere (Figure 4A,B). A broad band only observed for Cu#ZrO_2_ at around 1200 cm^–1^ (Figure 4B) is likely attributed to the CO_2_ molecules chemisorbed to the Cu surface.^31^

Further, we performed in situ Raman spectroscopy on Cu#ZrO_2_ using a home-made flowing system filled with a CO_2_-saturated aqueous electrolyte of 0.1 M KHCO_3_ (see the Supporting Information, Figure S1). Figure 4C,D shows the Raman spectra in a potential range from the OCP of 0.0 to −0.6 V vs RHE. A prominent band is recognized at 2886 cm^–1^ over the Cu#ZrO_2_ surface in the CO_2_ atmosphere at −0.2 V vs RHE (Figure 4D). This band is assigned to the C–H stretching of *O(CH)O*, which strongly correlates with the generation of FA.^32,33^ When the potential was more negative than −0.4 V, the peak intensity of *O(CH)O* at 2886 cm^–1^ was decreased, and no other intermediate species were seen in this region. These results suggest that the intermediate species were rapidly converted into FA and desorbed from the catalyst surface under application of high cathodic bias.^34,35^ The asymmetric stretching *CO_2_^–^ of adsorbed CO_2_ is observed at 1532 cm^–1^, while 1610 cm^–1^ is assigned to the O–C–O vibration of the adsorbed formate (Figure 4C). Summarizing this spectroscopic data, we propose a possible molecular scenario for the Cu#ZrO_2_ catalyst. The CO_2_ molecules that were physisorbed on the Zr^4+^ site to result in a reflectance band in DRIFTS 650 cm^–1^ (Figure 4B) were further transferred to the Cu^0^ site across the Cu–ZrO_2_ interface perimeter for further chemical adsorption (1200 cm^–1^ in DRIFTS, 1532 cm^–1^ in Raman). The protonation of adsorbed CO_2_ proceeds through sequential steps involving the formation of *O(CH)O* species (2886 cm^–1^ in Raman) and of the formate species that is identified as a band at 1610 cm^–1^ in Raman.

## 4 Conclusions

In conclusion, nanophase-separated Cu#ZrO_2_ was successfully obtained by internal oxidation of the Cu_51_Zr_14_ precursor alloy. Microscopic characterizations have demonstrated that the Cu#ZrO_2_ material consists of nanometer-thick lamellae of Cu metal and tetragonal ZrO_2_, leading to a stable and widespread Cu–ZrO_2_ interface. The Cu#ZrO_2_ material exhibits enhanced selectivity toward the electrocatalytic CO_2_-to-FA conversion due to the uniformly distributed catalytic sites as well as the abundant metal–oxide interface, which play an important role in amended reaction selectivity. DRIFTS and in situ Raman spectroscopy have shown that CO_2_ is adsorbed on the Zr^4+^ site and further protonated into *O(CH)O* over the neighboring Cu site to generate FA via a bifunctional interplay between Cu- and ZrO_2_ in Cu#ZrO_2_.

## Supporting Information Available

Additional experimental data, including experimental setup for in situ Raman measurements (PDF)
