# Supplementary material for: Nanophase-Separated Copper–Zirconia Composites for Bifunctional Electrochemical CO2 Conversion to Formic Acid
Source: ACS Appl Mater Interfaces. 2023 May 4;15(19):23299–305. doi: 10.1021/acsami.3c02874 (PMC10197065; doi:10.1021/acsami.3c02874)
Supplement: Supplementary file 1 — am3c02874_si_001.pdf [file am3c02874_si_001.pdf]

SUPPORTING INFORMATION

A Nanophase-Separated Copper-Zirconia Composite for Bifunctional Electrochemical CO_2 Conversion to Formic Acid

Anna Strijevskaya,[§,Δ] Akira Yamaguchi,[§] Shusaku Shoji,[†] Shigenori Ueda,[‡] Ayako Hashimoto,[‡,‖] Yu Wen,[‡,‖] Aufandra Cakra Wardhana,[§] Ji-Eun Lee,[♦] Min Liu,[○] Hideki Abe,[‡,■*] Masahiro Miyauchi.[§*]

[§] Department of Materials Science and Engineering, School of Materials and Chemical Technology, Tokyo Institute of Technology, Meguro, Tokyo, 152-8552, Japan.

[Δ] Uzbek-Japan Innovation Center of Youth, Tashkent, 100095, Uzbekistan.

[†] Department of Materials Science & Engineering, Cornell University, Ithaca, New York, 14853-1501, USA.

[‡] National Institute for Materials Science, Tsukuba, Ibaraki, 305-0044, Japan.

[‖] Graduate School of Pure and Applied Sciences, University of Tsukuba, Tsukuba, Ibaraki, 305-8571, Japan.

[♦] Biofunctional Catalyst Research Team, RIKEN Center for Sustainable Resource Science, Wako, Saitama, 351-0198, Japan.

[○] Hunan Joint International Research Center for Carbon Dioxide Resource Utilization, School of Physical and Electronics, Central South University, Changsha, 410083, Public Republic of China.

[■] Graduate School of Science and Technology, Saitama University, Saitama, 338-8570, Japan.

* Corresponding Authors' Email: mmiyauchi@ceram.titech.ac.jp
Abe.Hideki@nims.go.jp

Contents

1. *In situ* Raman spectroscopy flow cell set up (Figure S1)

2. Additional data of characterization and electrochemical CO_2 reduction properties

Figure S2. CV, LSV and 10 hours stability test of CO_2RR

Figure S3. Partial current density and Tafel analysis for formic acid generation

Figure S4. FE-SEM images of Cu#ZrO_2 before and after 3 hours of CO_2RR

Figure S5. STEM images of Cu#ZrO_2 after 30 min of CO_2RR reaction

Figure S6. XPS analysis of Cu#ZrO_2 after 5 hours of CO_2RR reaction

Table S1. Comparison with reported catalysts for CO2 RR to formate.

Table S2. Molar fraction of Zr element presented in Cu#ZrO_2 subjected to different duration of electrochemical CO_2 reduction conditions

1. *In situ Raman spectroscopy flow cell set up*.

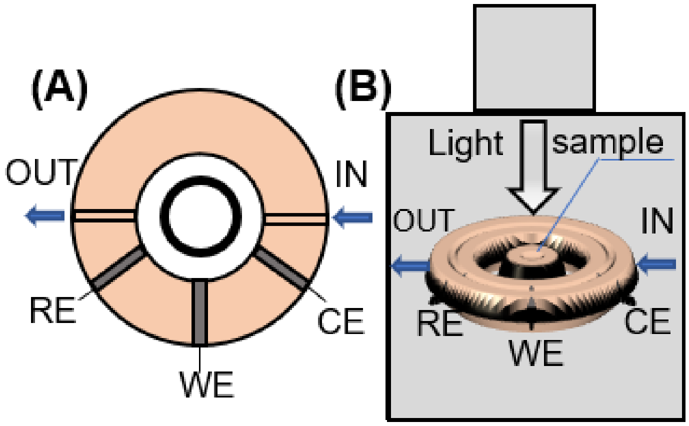

Figure S1. In situ Raman spectroscopy flow cell. A: top view, B: side view of the chamber

2. Additional data of characterization and electrochemical CO_2 reduction properties.

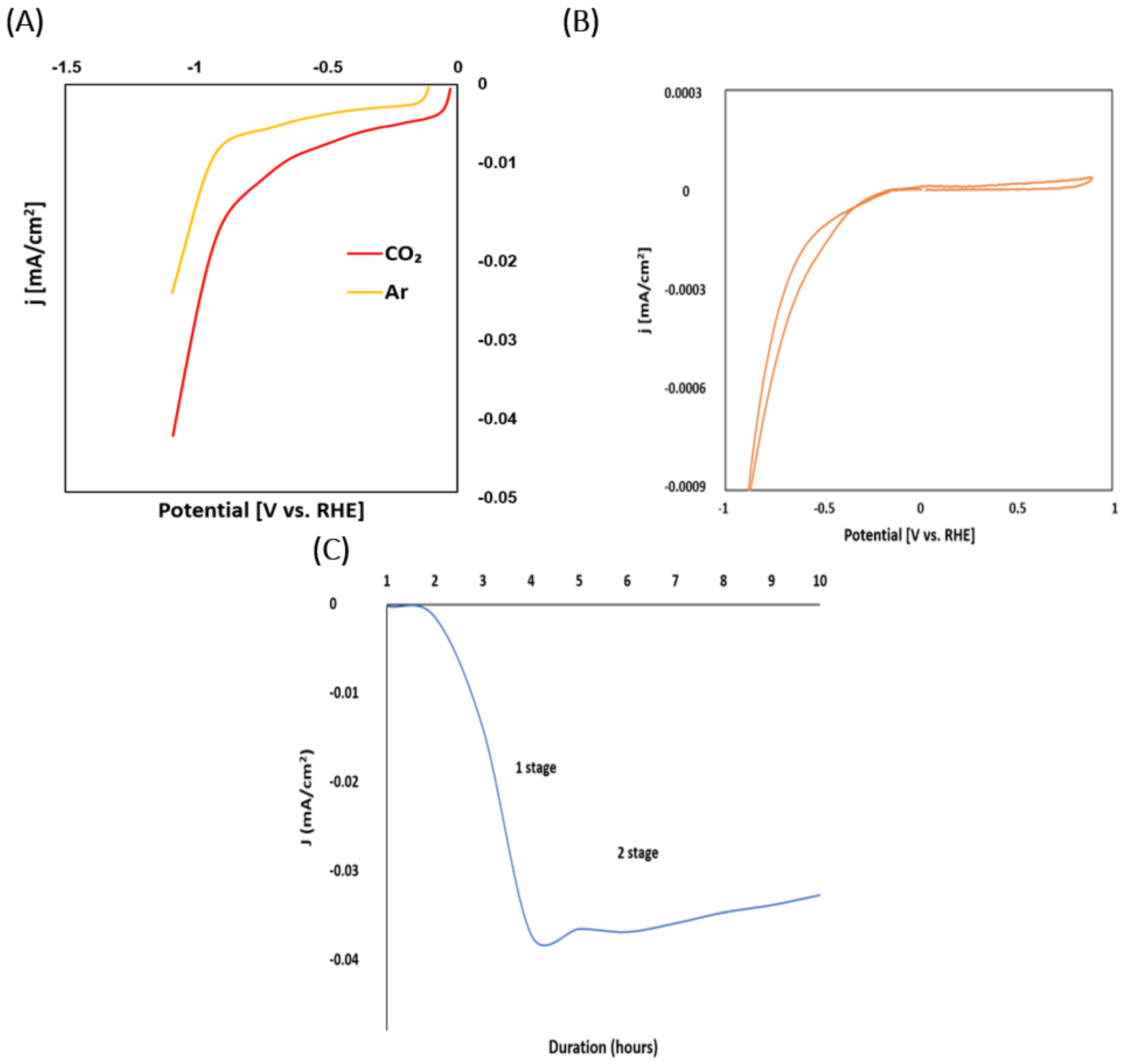

Figure S2. (A) Linear sweep voltammetry (LSV) scans were taken from 0.0 V to -1.1 V versus RHE at 20 mV/sec either in argon (Ar) or CO_2 atmosphere, respectively. The difference between the LSV profiles in a potential range more anodic than -0.3 V was attributed to a charging caused by adsorption of CO_2 molecules on the catalyst. (B) Cyclic voltammetry was taken in potential window from -1.0 V to 1.0 V vs. RHE at 100 mV/sec in 0.1M $KHCO_3$, purged with CO_2. (C) 10 hours stability test shows that CO_2 reduction over Cu#ZrO_2 occurs in two stages. In the first 4 hours, we could see the increase of current densities, attributed to the reaching of ZrO_2 to from active Cu sites. After 4 hours the current densities became stable.

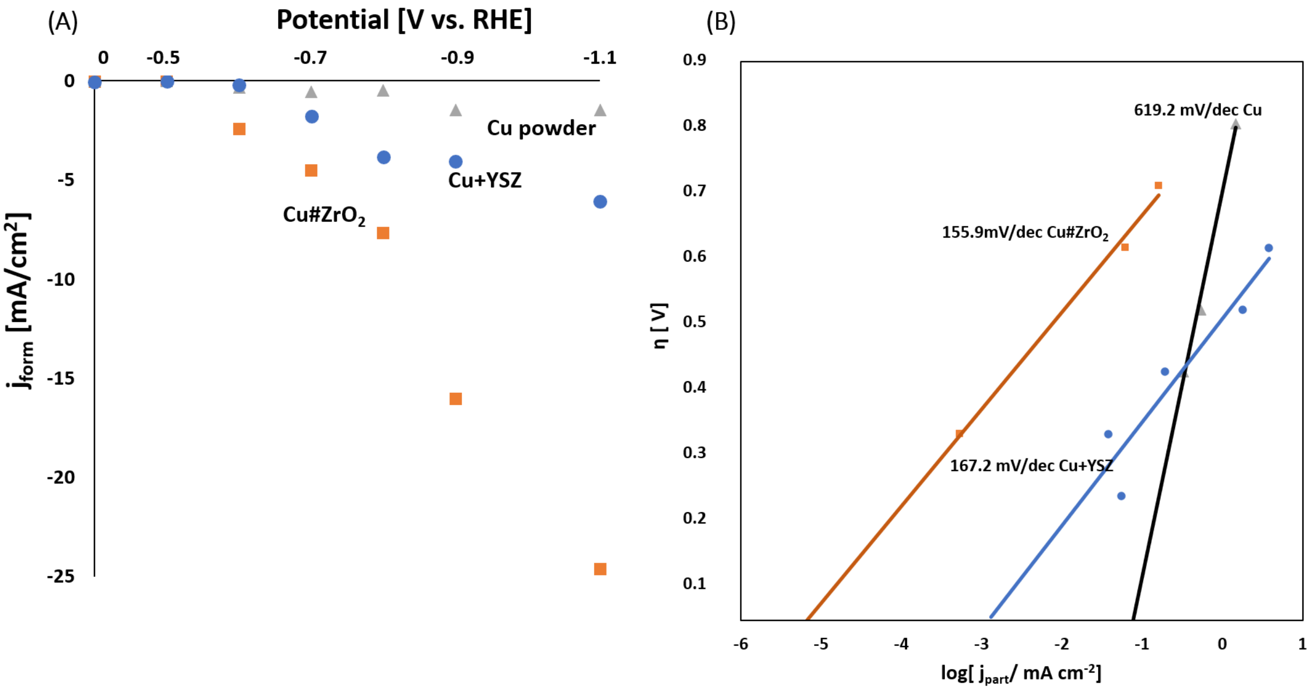

Figure S3. (A) Partial current density for FA measured for Cu#ZrO_2 (orange), Cu (grey) and the Cu+YSZ (blue) mixture based on 3 hours constant potential electrolysis in a range from −0.3 V to −1.1 V vs. RHE. Estimated electrochemical surface area of 10.9 m^2/g was used for calculation of current densities for Cu#ZrO_2; 214.7cm^2/g for Cu and Cu+ZrO_2 catalysts, respectively. (B) Tafel analysis for formic acid generation for Cu#ZrO_2 (orange), Cu+YSZ (blue), Cu (black)

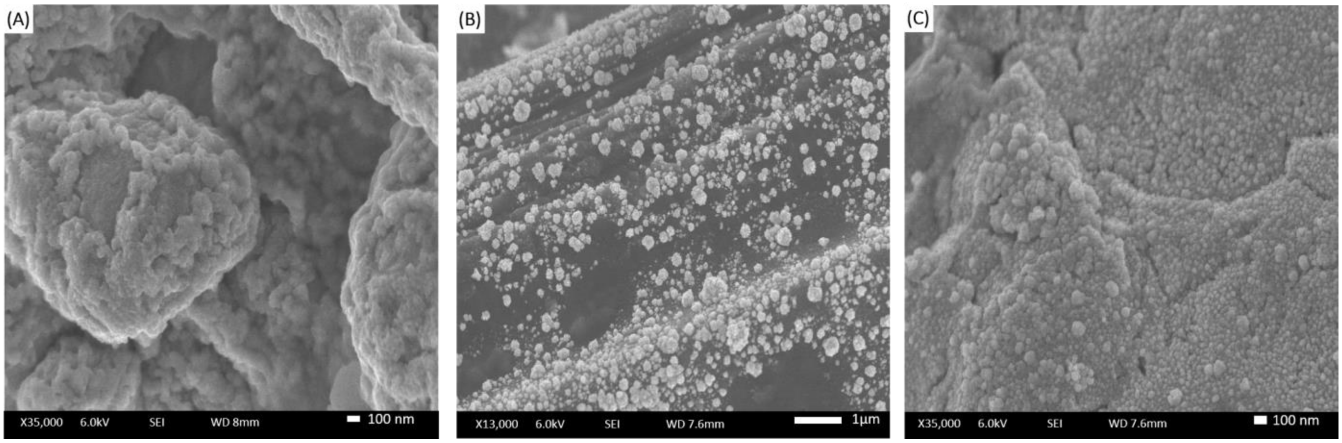

Figure S4. FE-SEM images of Cu#ZrO_2 catalyst on carbon paper before the CO_2 reduction reaction (A), low magnification image of Cu#ZrO_2 catalyst on carbon paper after 3 hours of the reaction (B), and the image of Cu#ZrO_2 catalyst after 3 hours of the reaction (C).

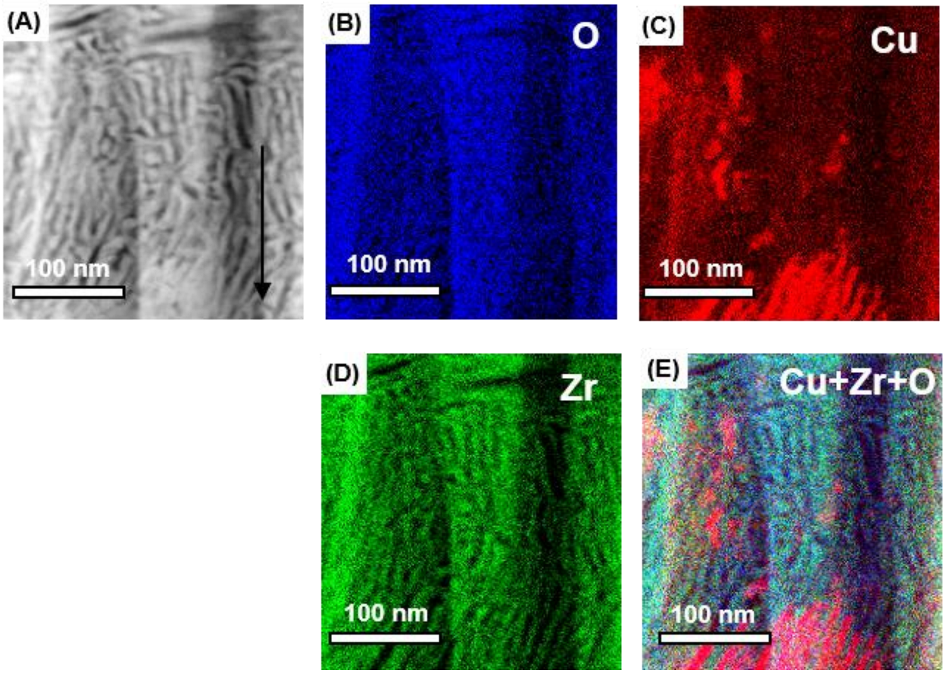

Figure S5. STEM- EDS images of Cu#ZrO_2 catalyst with a scale bar of 100 nm (A). Elemental mappings of O K (B), element distribution Cu K (C), Zr L (D), overlapped elemental mapping (E) after 30 min of electrochemical CO_2 reduction under -0.9V. Direction of arrow indicates the direction to the surface of the catalyst.

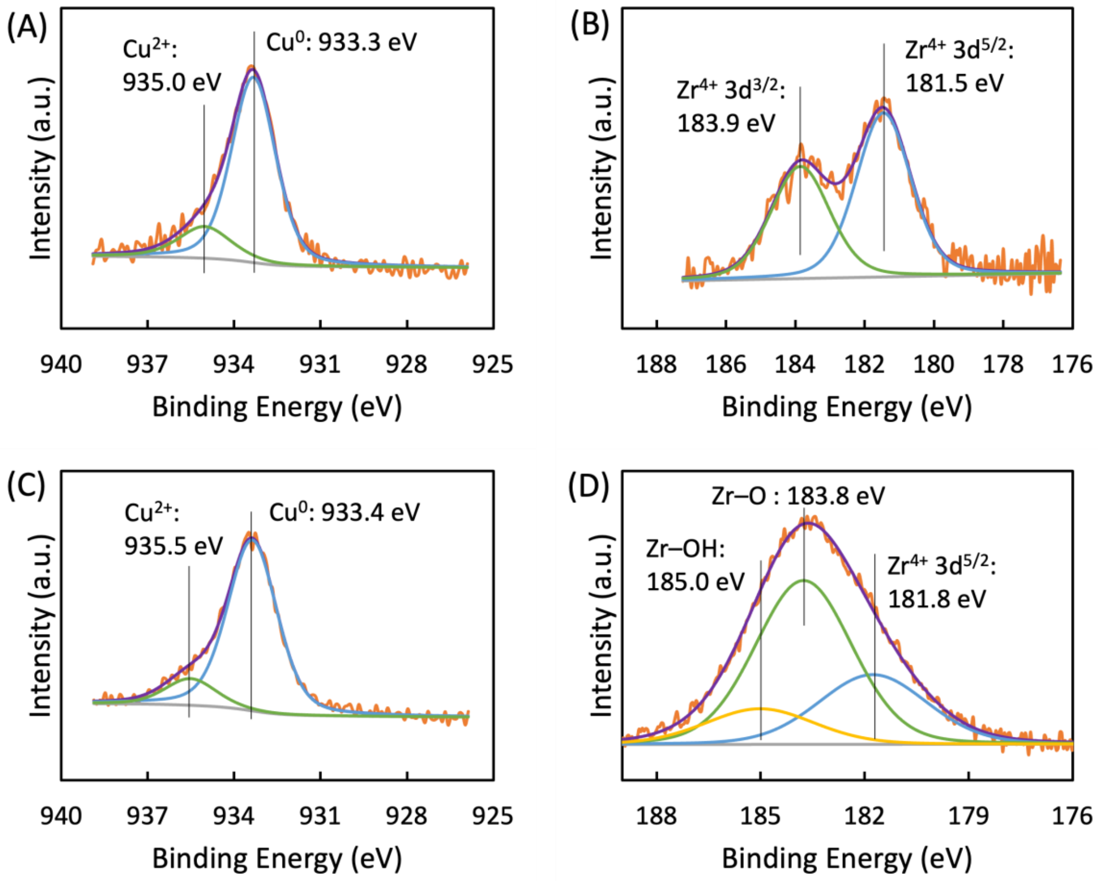

Figure S6. XPS spectra of Cu#ZrO_2 after: A, B- 5 min of electrochemical CO_2 reduction at -0.9 V RHE; C, D- after 5 hours.

Table S1. Faradaic efficiency of Cu#ZrO_2 for each product.

Products (gas or liquid)	FE%	Potential V vs RHE
HCOOH (l)	83.5	-0.9 V
H_2 (g)	17.1	
CO(g)	9.43	
CH_4 (g)	0.14	
C_2H_6 (g)	0.023	
Total FE: 110.2 ± 12 (%)		

Table S2. Comparison with reported catalysts for CO_2 RR to formate.

**Electrode**	**Electrolyte**	**Potential (V vs. RHE)**	**FE for formic acid (%)**	**Reference**
Cu#ZrO_2	0.1 M $KHCO_3$	-0.9	83.5	This work
Sn-Cu alloy	0.5 M KCl	-1.14	82.3	Ye K. et al[1]
Sn-doped Cu hollow fiber gas diffusion electrode	0.5 M $KHCO_3$	-1.2	78	Rabiee H. et al[2]
Cu-In dendric structure	0.1 M $KHCO_3$	-0.85	87.4	Shao J. et al[3]
BiO_n cluster	1 M $KHCO_3$	-1	82.4	Jiang X. et al[4]
CuBi	0.5 M $KHCO_3$	-0.97	94.4	Lou W. et al[5]
Bi-Sn aerogel	0.1 M $KHCO_3$	-1	93.9	Wu Z. et al[6]

Table S3. Molar fraction of Zr element presented in Cu#ZrO_2 subjected to different duration of electrochemical CO_2 reduction conditions.

Duration	Element	Mol. Fraction %
5 min	Zr	15.5
10 min	Zr	14.8
30 min	Zr	14.3
1 hour	Zr	13.2
2 hours	Zr	8.6
10 hours	Zr	7.2

References in Supporting Information

1. Ye K; Cao A.; Shao J.; Wang G.; Si R.; Ta N.; Xiao J. Synergy Effects on Sn-Cu Alloy Catalyst for Efficient CO2 Electroreduction to Formate with High Mass Activity. *Sci. Bull.* **2020,** *65*, 711-719.
2. Rabiee H.; Zhang X.; Ge L.; Hu Sh.; Li M.; Smart S.; Zhu Z.; Yuan Z. Tuning the Product Selectivity of the Cu Hollow Fiber Gas Diffusion Electrode for Efficient CO2 Reduction to Formate by Controlled Surface Sn Electrodeposition. *ACS Appl. Mater. Interfaces*, 2020, *12*, 21670-21681.
3. Shao J.; Wang Y.; Gao D.; Ye K.; Wang Q.; Wang G. Copper-Indium Bimetallic Catalysts for the Selective ElectroChemical Reduction of Carbon Dioxide. *Chinese J. Catal.* 2020, 41, 1393-1400.
4. Jiang X.; Lin L.; Rong Y.; Li R.; Jiang Q.; Yang Y.; Gao D. Boosting CO2 Electroreduction to Formate via Bismuth Oxide Clusters. Nano Res. **2022**, 1-8.
5. Lou W. J. Peng L.; He R.; Liu Y.; Qiao J. CuBi Electrocatalysts Modulated to Grow on Derived Copper Foam for Efficient CO2-to-Formate Conversion. Colloid. Interface Sci. **2022**, 606, 994-1003.
6. Wu Z.; Wu H.; Cai W.; Wen Z.; Jia B.; Wang L.; Jin W.; Ma T. Engineering Bismuth–Tin Interface in Bimetallic Aerogel with a 3D Porous Structure for Highly Selective Electrocatalytic CO2 Reduction to HCOOH. Angew. Chem. **2021**, 133, 12662-12667.
